# Production of High-Purity Anhydrous Nickel(II) Perrhenate for Tungsten-Based Sintered Heavy Alloys

**DOI:** 10.3390/ma10040448

**Published:** 2017-04-24

**Authors:** Katarzyna Leszczyńska-Sejda, Grzegorz Benke, Dorota Kopyto, Tomasz Majewski, Michał Drzazga

**Affiliations:** 1 Institute of Non Ferrous Metals (IMN), Hydrometallurgy Department, Sowińskiego 5, 44-100 Gliwice, Poland; grzegorzb@imn.gliwice.pl (G.B.); dorotak@imn.gliwicer.pl (D.K.); michald@imn.gliwice.pl (M.D.); 2 Military University of Technology in Warsaw (WAT), Kaliskiego 2, 00-908 Warszawa 49, Poland; tomasz.majewski@wat.edu.pl

**Keywords:** rhenium, nickel, nickel(II) perrhenate, Re-Ni powder, tungsten-based sintered heavy alloys

## Abstract

This paper presents a method for the production of high-purity anhydrous nickel(II) perrhenate. The method comprises sorption of nickel(II) ions from aqueous nickel(II) nitrate solutions, using strongly acidic C160 cation exchange resin, and subsequent elution of sorbed nickel(II) ions using concentrated perrhenic acid solutions. After the neutralization of the resulting rhenium-nickel solutions, hydrated nickel(II) perrhenate is then separated and then dried at 160 °C to obtain the anhydrous form. The resulting compound is reduced in an atmosphere of dissociated ammonia in order to produce a Re-Ni alloy powder. This study provides information on the selected properties of the resulting Re-Ni powder. This powder was used as a starting material for the production of 77W-20Re-3Ni heavy alloys. Microstructure examination results and selected properties of the produced sintered heavy alloys were compared to sintered alloys produced using elemental W, Re, and Ni powders. This study showed that the application of anhydrous nickel(II) perrhenate in the production of 77W-20Re-3Ni results in better properties of the sintered alloys compared to those made from elemental powders.

## 1. Introduction

Rhenium is a precious metal, which is recovered during molybdenum and copper production processes [1]. Due to its specific properties, rhenium can be applied in the production of tungsten-based sintered heavy alloys [2]. Rhenium dopant improves the properties of tungsten-based heavy alloys by enhancing their hardness and strength as well as decreasing their ductility [3]. The material deformation mechanism changes due to the rearrangement of oxides and carbides in the alloy microstructure [4]. This results in the spherical form of tungsten oxides, and their uniform distribution throughout the volume of the alloy. Thanks to these properties, the alloys can be used for specialty-product manufacturing, e.g., elements of aircrafts and armaments, as well as thermocouples, catalysts, and some kinds of electrical contacts [5,6]. Tungsten-based sintered heavy alloys are usually produced from metal powders of individual components [7]. Their contents are selected to guarantee the required final product composition. Rhenium compounds, usually ammonium perrhenate instead of metallic rhenium, are used in the production of rhenium-containing sintered heavy alloys [8,9]. The other alloy components, i.e., nickel and tungsten, are introduced as elemental powders.

Previously-applied rhenium sources may be substituted by a new one—anhydrous nickel(II) perrhenate. This compound contains two components of the tungsten-based sintered heavy alloys, rhenium and nickel [10]. 

There are not many publications concerning nickel(II) perrhenate; though the first one dates back to the 1930s [11]. Over the years, only a few articles describing its production, physicochemical properties, and applications have been published [12,13,14,15]. It is reported that nickel(II) perrhenate may be obtained by the neutralization of perrhenic acid with nickel(II) carbonate or hydroxide [16], as well as from nickel salt solutions using the ion-exchange method [17]. In the production of tungsten-based heavy alloys, elemental metal powders can be replaced by Re-Ni alloy ones, which may be obtained through the reduction of Ni(ReO_4_)_2_. They should provide a better powder mixture homogenization, sintering process cost reduction (shorter sintering time, lower process temperature), diffusion acceleration during sintering, reduction of the individual powders oxidation, and homogeneous sintered alloys of a better uniformity than in the case of the metal powders mixture.

## 2. Experimental Section

### 2.1. Materials

The studies were carried out using strongly acidic C160 cation-exchange resin in the hydrogen form (Purolite, Gdynia, Poland). Pure perrhenic acid, produced using the ion-exchange method at IMN [18,19] from catalytically-pure ammonium perrhenate (Innovator, Gliwice, Poland), was used as a rhenium source. The acid contained 400–900 g/dm^3^ Re and Ca < 0.0001%, K < 0.0005%, Mg < 0.0001%, Cu < 0.0001%, Na < 0.0001%, Mo < 0.0001%, Ni < 0.0001%, Pb < 0.0001%, Fe < 0.0001%, NH_4_^+^ < 0.0002%, Bi < 0.0001%, Zn < 0.0001%, W < 0.0001%, As < 0.0001%, and Al < 0.0001%. In the experimental studies, nickel(II) oxide (Alfa Aesar, Chemat, Gdańsk, Poland), nitric acid, and aqueous hydrogen peroxide solution (Avantor, Gliwice, Poland) were also used. All the reagents were of analytical grade purity. Distilled water (<2 μS/cm) was used in the experiments. Elementary powders applied in the sintering process were: tungsten powder (99.98%, mean particle diameter 2.45 μm, Węgliki Spiekane “Baildonit”, Katowice, Poland), nickel powder Type 123 (99.9%, mean particle size 3.7 μm, Vale, Toronto, ON, Canada) and rhenium powder (99.89%, mean particle size 5.8 μm) made by WAT.

### 2.2. Nickel(II) Perrhenate Production Using the Ion-Exchange Method

A new method of anhydrous nickel(II) perrhenate synthesis, as an alternative to the classical one, was developed. It was composed of two stages: Ni^2+^ ion sorption and their elution using aqueous perrhenic acid solution:

sorption:  2[cationite]-H^+^ + Ni^2+^ → [cationite]_2_-Ni^2+^ + 2H^+^

elution:  [cationite]_2_-Ni^2+^ + 2HReO_4_ → 2[cationite]-H^+^ + Ni(ReO_4_)_2_

**Figure 1:**
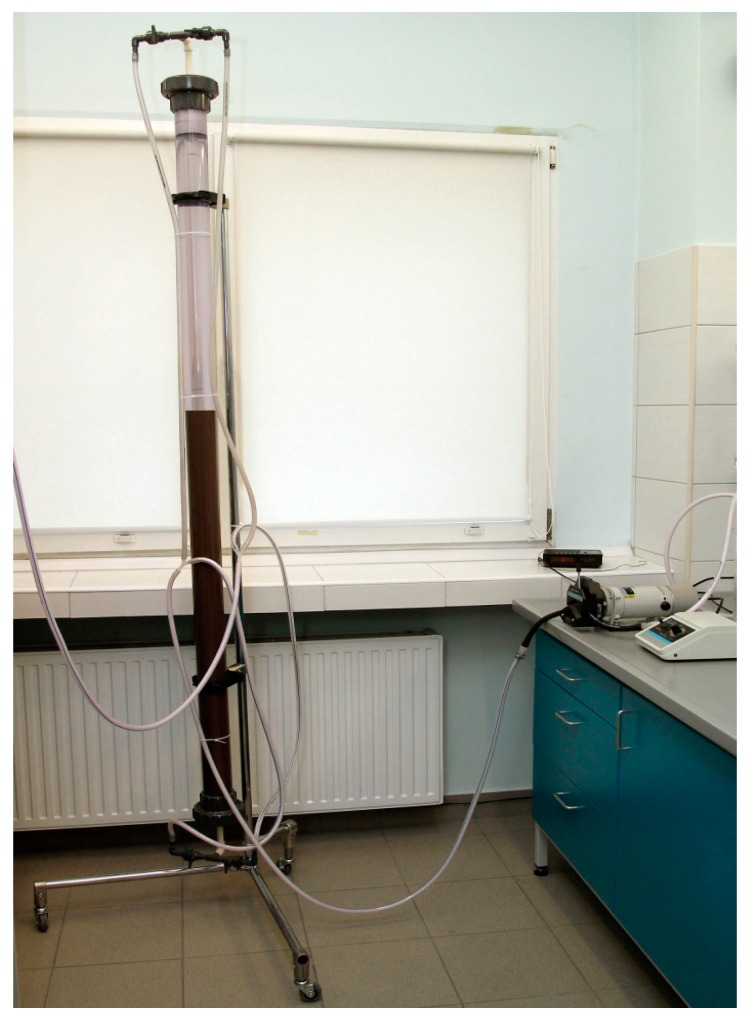
Ion-exchange column for the production of Ni(ReO_4_)_2_.

The process was conducted in a specially-designed ion-exchange column, 0.035 m in diameter and 1.5 m in height, made of transparent RPVC (Reinforced PolyVinyl Chloride), resistant to strong oxidants. The column was equipped with two heads, an upper and a lower, to allow solution flow in both directions—down and up. The ion-exchanger bed was filled to 80% of the column volume. The ion-exchange system also included a peristaltic pump (Masterflex L/S), and is shown in Figure 1.

Nickel(II) nitrate was dissolved in water and then filtered to obtain a clear solution with a concentration of 5.0 g/dm^3^ Ni. This solution was passed down the column through a C160 ion-exchange bed at a flow rate of 2.5 dm^3^/h, until the concentration of nickel ions in the effluent exceeded 1.0 g/dm^3^. Then, the saturated ion-exchange resin was washed at a rate of 5.0 dm^3^/h, with 2 dm^3^ of water, down the column until a neutral pH was reached. The resulting post-washing solution was reverted to the nickel(II) nitrate solution preparation stage. The washed ion-exchanger was eluted with 1 dm^3^ of perrhenic acid solution, containing 420 g/dm^3^ Re, down the column, at a flow rate of 0.25 dm^3^/h. The eluate was collected in two batches; the first contained less than 0.01 g/dm^3^ Re, while the second contained higher concentrations of nickel and rhenium. After the elution, the ion-exchanger was washed with water until a neutral pH was reached. After the elution, the column was washed and the post-washing solutions were also collected in two batches. The first batch was reverted to the eluent preparation stage (HReO_4_), while the second was directed to the nickel(II) nitrate preparation stage. 

Ten cycles of the entire process were performed. After the tenth cycle, the ion-exchanger was regenerated using 1 dm^3^ of 32% nitric(V) acid. The regeneration was carried out up to the column, at a flow rate of 2.5 dm^3^/h. Then, the ion-exchanger was washed twice with water—first, at a flow rate of 5.0 dm^3^/h with 2 dm^3^ water, up to the column, and then using ca. 3 dm^3^ of water at a flow rate of 2.5 dm^3^/h, down to the column, until a neutral eluate pH was reached. After the regeneration, the ion-exchanger was used in eight further process cycles. The tests after the regeneration were carried out under the same conditions.

High-purity nickel(II) oxide was added for 2 h, in portions, to the second batch of the post-elution solution, i.e., the one with higher nickel and rhenium concentrations. The dissolution was carried out below 80 °C, until solution neutralization was achieved. Then, the solution was filtered to remove the remaining solids and was subsequently evaporated below 80 °C until dry. The separated tetrahydrate form of crude nickel(II) perrhenate was dissolved in 10% aqueous hydrogen peroxide solution and stirred at room temperature for 30 min. The solution was then filtered and subsequently evaporated—the operation was repeated twice. Finally, the obtained hydrated nickel(II) perrhenate was dried at 160 °C to obtain its anhydrous form. 

The sorption efficiency of nickel ions (*W_Ni_*), elution efficiency of nickel ions (*E_Ni_*), and saturation degree of the ion-exchanger with nickel ions (*S_Ni_*) were calculated as follows:

(1) $$W_{Ni}=\frac{m_{Ni}-C_{Ni}\cdot V_{rs}}{m_{Ni}}\cdot 100\%$$

(2) $$E_{Ni}=\frac{C_{NiE}\cdot V_{re}}{m_{Ni}-C_{Ni}\cdot V_{rs}}\cdot 100\%$$

(3) $$S_{Ni}=\frac{m_{Ni}-C_{Ni}\cdot V_{rs}}{m_{j}}\cdot 100\%$$

where *m_Ni_*—nickel mass in the initial solution (g); *C_Ni_*—nickel concentration in the post-sorption solution (g/dm^3^); *V_rs_*—solution volume after sorption (dm^3^); *C_NiE_*—nickel concentration in the post-elution solution (g/dm^3^); *V_re_*—solution volume after elution (dm^3^); and *m_j_*—ion exchanger mass (g).

### 2.3. Reduction of Anhydrous Nickel(II) Perrhenate

Reduction annealing was performed by placing 100-g batches of anhydrous nickel(II) perrhenate in a tubular furnace (RO 13.5) under a dissociated ammonia atmosphere with a –30 °C dew point. The study was carried out in the temperature range of 700–900 °C for 1–2 h. 

### 2.4. Production of 77W-20Re-3Ni Sintered Alloys

The mixture of 252.4 g of tungsten powder and 76 g of the reduced Re-Ni powder was homogenized in a planetary ball mill. The powders were compacted under a pressure of 300 MPa, using the cold isostatic pressing (CIP) method, to produce cylindrical rods. Sintering of the compacts was carried out in two stages. First, preliminary sintering in the solid phase using a tubular furnace was conducted in an atmosphere of dissociated ammonia with a −30 °C dew point, at 1150 °C for one hour. Then, the compacts were cooled down in a furnace cooler to an ambient temperature. Second, final sintering with the presence of the liquid phase was performed in a horizontal vacuum furnace at 1520 °C and 10^−2^ hPa for 2 h. The sintered alloys were cooled to 1420 °C at a rate of 5 °C/min. Further cooling was carried out at a rate of 15 °C/min. 

### 2.5. Analytical Methods

The analyses of nickel(II) perrhenate and Re-Ni powder were performed at the Institute of Non Ferrous Metals (IMN) Department of Analytical Chemistry. Rhenium content was analyzed using thin layer X-ray fluorescence spectrometry, using a fluorescent X-ray spectrometer (ZSX Primus, Rigaku, Tokyo, Japan). Nickel content was determined using the FAAS (Flame Atomic Absorption Spectroscopy) method. Concentration of some pollutants (As, Cu, Mg, Zn, Ca, Fe, Mo, Pb, Na, Bi, K, Co, Mn, Cr, and Al) were determined using the following instrumental techniques: GFAAS (graphite furnace atomic absorption spectroscopy with graphite cells, Z-2000, HITACHI, Tokyo, Japan), ICP-OES (Inductively Coupled Plasma—Optical Emission Spectrometers; ULTIMA 2, HORIBA Jobin-Ivon, Kyoto, Japan), and ICP MS (Inductively Coupled Plasma—Mass Spectroscopy; Nexion, PerkinElmer, Waltham, MA, USA). Carbon content was determined using the IR (InfraRed) method after the combustion of the sample in an oxygen stream using a CS2000 analyzer (Eltra, Haan, Germany). Rhenium and nickel concentrations in the solutions, generated during Ni(ReO_4_)_2_ synthesis, were determined using FAAS, using a THERMO atomic absorption spectrometer SOLAAR S4 (Thermo Electron Corporation, Waltham, MA, USA) equipped with a flame module and deuterium background correction. 

The X-ray phase analysis of Re-Ni powder was performed at the Tele and Radio Research Institute in Warsaw. Examination of the alloy phase compositions was conducted by diffraction measurements (XRD) using a D500 diffractometer (Siemens, Karlsruhe, Germany) equipped with a semiconductor Si[Li] detector. The applied KαCu radiation was of an average wavelength of λ = 1.5418 Å, the lamp parameters were U = 40 kV and J = 30 mA, and the collimator aperture was 1°/1°/1°/0.05°. The measurements were performed stepwise in the 2Θ range between 20° and 70°, at 0.05° increments, using a 10-s counting time.

## 3. Results and Discussion

### 3.1. Ion-Exchange Method for Nickel(II) Perrhenate Production

The study consisted of 10 process cycles before the ion-exchange resin regeneration, and eight cycles after regeneration. It was found that the application of the previously-described conditions (see Section 2.2) resulted in high sorption efficiency, exceeding 99%. Additionally, the saturation degree of the resin with nickel was also high, and amounted to 5.8%. About 10 dm^3^ of the solution was treated in a single cycle. It was established that the ion-exchanger, after the regeneration, also guaranteed a stable course for the sorption process—for each cycle, the sorption efficiency exceeded 99.7%. The results of nickel ion sorption tests, using the ion-exchange resin, before and after the regeneration, are shown in Table 1 and Table 2, respectively.

The elution efficiencies of nickel ions (Table 3 and Table 4) were in the range of 53.4%–99.7%, and gradually declined in subsequent cycles. A very large drop in the elution efficiency in the 9th cycle was observed. Elution test results, after the resin regeneration, indicated that this operation is necessary for stable operation of the ion-exchanger. Elution efficiencies after the regeneration were higher, more stable, and equal to at least 99.0%. 

The produced nickel(II) perrhenate tetrahydrate had elevated concentrations of sodium, magnesium, and calcium, harmful contaminants, as well as cobalt and iron, components often present in sintered heavy alloys; other contaminant levels were as expected. The content of zinc, bismuth, copper, and arsenic did not exceed 0.0002% in each studied sample. The lead, chromium, aluminum, molybdenum, and potassium levels in the separated compound were below 0.0005%, and carbon was <0.002%. The contents of impurities in the selected nickel(II) perrhenate tetrahydrate samples are shown in Table 5. 

Significant increases in calcium, magnesium, sodium, cobalt, and iron levels in the separated Ni(ReO_4_)_2_ were observed for the subsequent cycles of the process. Other element concentrations, such as carbon, molybdenum, manganese, zinc, potassium, copper, bismuth, aluminum, chromium, and arsenic, remained unchanged. 

The first purification stage allowed the removal of all the calcium, iron, cobalt, and magnesium, as well as ca. 90% of the sodium. In the next purification step, the remaining sodium content was removed. The studies resulted in the development of an anhydrous nickel(II) perrhenate production method with the following composition: 10.5% Ni, 66.5% Re and <0.0005% Ca, <0.0005% K, <0.0005% Mg, <0.0005% Cu, <0.0005% Al, <0.0005% Cr, <0.0005% Mn, <0.0005% Na, <0.0005% Mo, <0.0005% Co, <0.0005% Pb, <0.0005% Fe, <0.002% C, <0.0002% Zn, <0.0002% As, and <0.0002% Bi. About 50 kg of the substance, with a standardized composition, was produced using this method. The manufactured anhydrous nickel(II) perrhenate was used in the investigation of tungsten-based, sintered heavy alloy production.

### 3.2. Reduction of Anhydrous Nickel(II) Perrhenate

The produced Re-Ni powder was examined using X-ray phase analysis, mass loss determination from the thermal reduction process, hydrogen loss measurements, metallographic observations of the produced powders, bulk and tapped density measurements, powder granulometry, and the determination of Re, Ni, and impurity content using chemical analysis.

**Figure 2:**
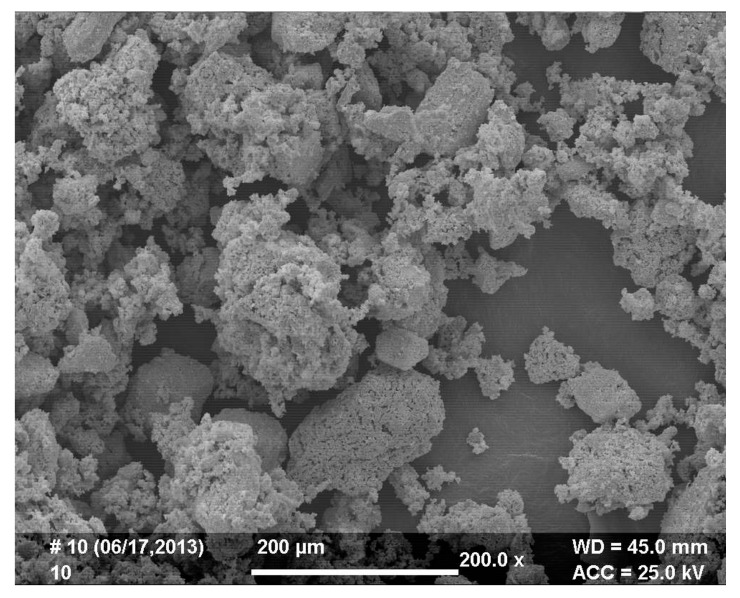
Microstructure of the Re-Ni powder, obtained after 1 h reduction at 800 °C (200× magnification).

**Figure 3:**
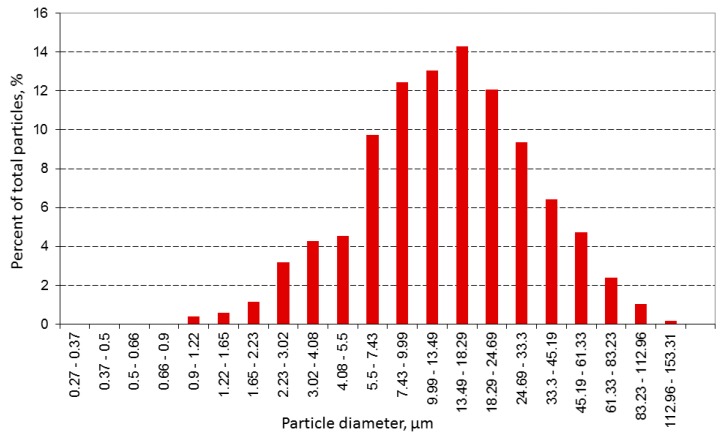
Particle size distribution of Re-Ni alloy powder: Thermal reduction at 800 °C for 1 h.

It was found that complete material reduction can be achieved for nickel(II) perrhenate; thermal reduction was conducted at 800 °C for one hour. Figure 2 and Figure 3 show the microstructure and size distribution of the reduced powder, respectively.

**Figure 4:**
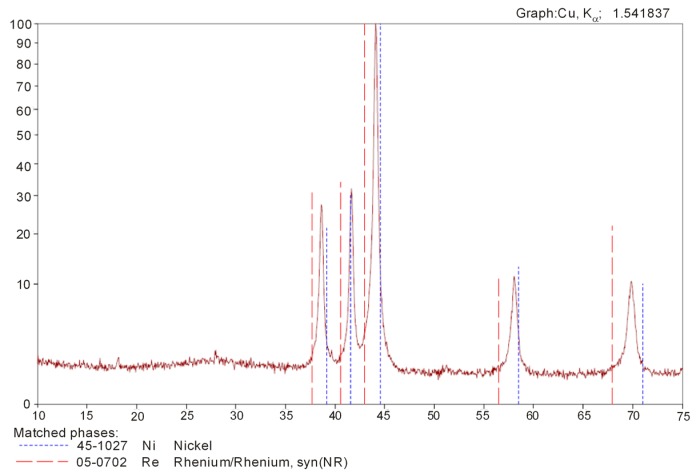
Diffractogram of Re-Ni alloy powder produced by Ni(ReO_4_)_2_ reduction at 800 °C for 1 h.

It was determined that powder particles formed agglomerates after reduction. Therefore, the powder should have been comminuted, e.g., using a ball mill, before its application in sintered alloy manufacturing. Figure 4 presents the diffraction pattern of the Re-Ni powder phase analysis. The thermal reduction process resulted in a completely alloyed powder, which contained: 13.6% Ni, 86.4% Re, <0.0005% Ca, <0.0005% K, <0.0005% Mg, <0.0005% Cu, <0.0005% Al, <0.0005% Cr, <0.0005% Mn, <0.0005% Na, <0.0005% Mo, <0.0005% Co, <0.0005% Pb, <0.0005% Fe, <0.002% C, <0.0002% Zn, <0.0002% As, and <0.0002% Bi. 

### 3.3. Examination of 77W-20Re-3Ni Sintered Alloys

**Figure 5:**
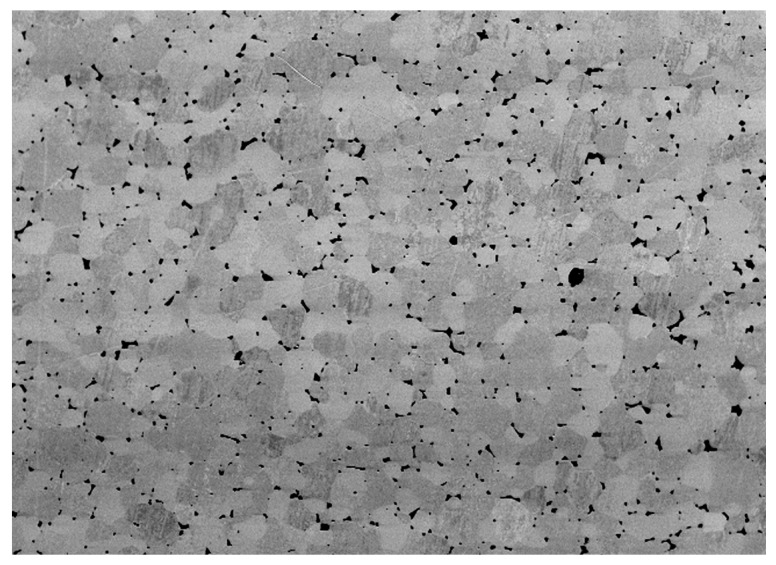
Microstructure image of the 77W-20Re-3Ni alloy (500× magnification).

**Figure 6:**
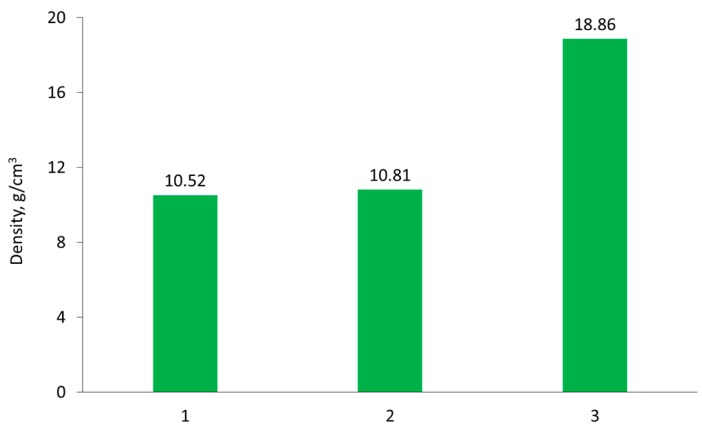
Densities of the compact and sintered 77W-20Re-3Ni alloy: 1—compact; 2—alloy after initial sintering; 3—alloy after vacuum sintering at 1520 °C for one hour.

Selected examination results of 77W-20Re-3Ni sintered alloys, obtained according to the previously described procedure (see Section 2.4) are presented below. Figure 5 shows the microstructure of the produced sintered alloy. The particles of the high-melting phase and a small area of the bonding phase on the particle edges can be observed. Figure 6 illustrates the density changes of the tested samples after various stages of the process.

**Figure 7:**
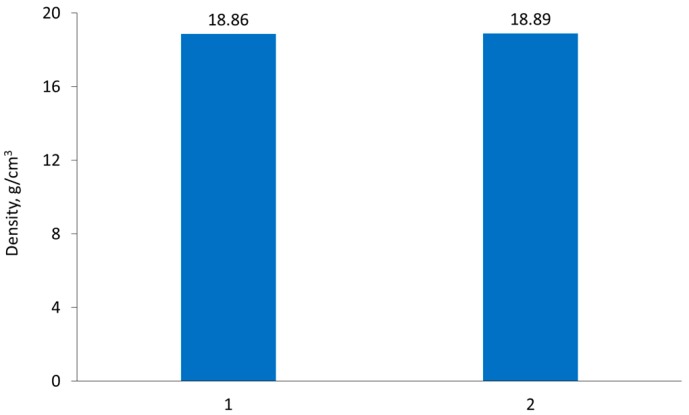
Chart of 77W-20Re-3Ni alloy densities after vacuum sintering: 1—at 1520 °C for one hour; 2—at 1650 °C for one hour.

The influence of vacuum-sintering temperature on final product density was also determined. Figure 7 shows that the temperature increase, from 1520 °C to 1650 °C, had no influence on density. Therefore, it may be concluded that a sintering temperature of 1520 °C provides sufficient process conditions for 77W-20Re-3Ni sintered alloy. 

Table 6 presents selected properties of the sintered alloys, produced using Re-Ni alloy powder, compared to an alloy made from elemental powders (metallic rhenium obtained using ammonium perrhenate thermal reduction, as was used in comparative studies). The results presented in Table 6 confirm that the application of Re-Ni alloy powder results in a sintered alloy with lower porosity, higher strength, and increased ductility, compared to that produced with elemental powders.

## 4. Conclusions

Based on the obtained results, the following conclusions can be drawn:

1. Application of the ion-exchange method, using a strongly acidic C160 cation-exchange resin, allows a cyclic production of high-purity anhydrous nickel(II) perrhenate with the following composition: 10.5% Ni, 66.5% Re, <0.0005% Ca, <0.0005% K, <0.0005% Mg, <0.0005% Cu, <0.0005% Al, <0.0005% Cr, <0.0005% Mn, <0.0005% Na, <0.0005% Mo, <0.0005% Co, <0.0005% Pb, <0.0005% Fe, <0.002% C, <0.0002% Zn, <0.0002% As, and <0.0002% Bi.
2. Sorption and elution efficiencies greater than 99.0% of nickel ions can be maintained by regeneration of the ion-exchanger with 32% nitric(V) acid, which was performed every eight cycles.
3. The thermal reduction of anhydrous nickel(II) perrhenate under the adopted conditions allows the production of Re-Ni alloy powder containing: 13.6% Ni, 86.4% Re, <0.0005% Ca, <0.0005% K, <0.0005% Mg, <0.0005% Cu, <0.0005% Al, <0.0005% Cr, <0.0005% Mn, <0.0005% Na, <0.0005% Mo, <0.0005% Co, <0.0005% Pb, <0.0005% Fe, <0.002% C, <0.0002% Zn, <0.0002% As, and <0.0002% Bi.
4. Sintered heavy alloys manufactured using Re-Ni alloy powder display better physical and mechanical properties than materials sintered using elemental powders and rhenium, obtained using ammonium perrhenate thermal reduction.

## Figures and Tables

**Table 1:** Nickel(II) ions sorption results—10 cycles before the regeneration.

Cycle	Volume of Post-Sorption Effluent, dm^3^	Nickel Concentration in Post-Sorption Effluent, g/dm^3^	Nickel(II) Ions Sorption Efficiency, %
I	10.5	0.8	99.8
II	11.0	1.1	99.7
III	10.2	1.2	99.7
IV	9.6	1.3	99.7
V	10.6	0.7	99.8
VI	10.5	0.9	99.8
VII	10.4	1.1	99.7
VIII	10.8	1.2	99.7
IX	11.0	0.8	99.8
X	10.2	0.5	99.9

**Table 2:** Nickel(II) ions sorption results—eight cycles after the regeneration.

Cycle	Volume of Sorption Effluent, dm^3^	Nickel Concentration in Sorption Effluent, g/L	Nickel(II) Ions Sorption Efficiency, %
I	10.4	0.4	99.9
II	11.0	0.3	99.9
III	11.2	0.5	99.8
IV	10.9	0.8	99.8
V	10.5	0.8	99.8
VI	11.2	0.8	99.8
VII	10.2	0.9	99.8
VIII	10.4	1.1	99.7

**Table 3:** Nickel(II) ions elution results—10 cycles before the regeneration.

Cycle	Volume of Elution Effluent, dm^3^	Rhenium Concentration in Elution Effluent, g/dm^3^	Nickel Concentration in Elution Effluent, g/dm^3^	Nickel(II) Ions Elution Efficiency, %
I	1.1	380	32.3	71.2
II	1.1	375	41.1	90.6
III	1.3	313	36.5	95.1
IV	1.2	340	38.7	93.1
V	1.3	320	34.6	90.1
VI	1.1	367	40.1	88.4
VII	1.2	341	37.2	89.5
VIII	1.2	340	38.2	91.9
IX	1.3	312	20.5	53.4
X	1.3	310	21.2	55.2

**Table 4:** Nickel(II) ions elution results—eight cycles after the regeneration.

Cycle	Volume of Elution Effluent, dm^3^	Rhenium Concentration in Elution Effluent, g/dm^3^	Nickel Concentration in Elution Effluent, g/dm^3^	Nickel(II) Ions Elution Efficiency, %
I	1.1	380	45.2	99.5
II	1.1	378	45.1	99.3
III	1.1	370	45.0	99.1
IV	1.1	380	45.0	99.2
V	1.2	350	41.2	99.0
VI	1.1	370	45.1	99.4
VII	1.0	410	49.7	99.6
VIII	1.1	380	45.2	99.7

**Table 5:** Concentration of the selected impurities in the produced hydrated nickel(II) perrhenate.

Cycle	Na	Ca	Mg	Co	Fe
before regeneration, %					
I	0.050	0.004	0.002	0.006	0.008
V	0.060	0.005	0.003	0.006	0.010
VII	0.085	0.007	0.006	0.007	0.012
X	0.090	0.012	0.012	0.009	0.015
after regeneration, %					
I	0.012	0.001	0.001	0.004	0.004
V	0.025	0.002	0.001	0.004	0.006
VIII	0.040	0.002	0.002	0.005	0.006

**Table 6:** Selected properties of 77W-20Re-3Ni heavy alloy.

Alloys Composition, % Mass.	Density, g/cm^3^	Porosity, %	Yield Point, MPa	Compressive Strength, MPa	Unit Shortening, %	Hardness HRC
77W-20Re-3Ni *	18.86	0.38	1683.5	2408	18.6	52
77W-20Re-3Ni **	18.61	1.89	1453.0	2310	14	48

Note: * alloy produced from alloy powder Re-Ni; ** alloy produced from elementary powders.
